# ‘Letting Go’: delegating responsibility for non-clinical tasks in a telehealth service

**Published:** 2012-06-15

**Authors:** Claire Elizabeth Hurlin, Sarah Rees, Leo Lewis

**Affiliations:** Hywel Dda Health Board, UK; Hywel Dda Health Board, UK; Hywel Dda Health Board, UK

**Keywords:** partnership, comprehensive, timely telehealth service

## Abstract

**Introduction:**

The implementation of telehealth into the delivery of chronic conditions management within Hywel Dda Health Board has provided an opportunity to enhance close working relationships with Carmarthenshire County Council’s well-established telecare team. The responsibilities of the telecare team were initially limited to the installation and removal of telehealth devices in patients’ homes and training on its use but as the use of telehealth has widened, an increasing number of non-clinical tasks, several of which were previously undertaken by clinical staff, have been delegated to members of the telecare team and linked to the monitoring centre. In addition, all the tasks associated with managing and administering the patients on the telehealth system backend are undertaken by the chronic conditions management administrative support team within the Health Board.

**Aims and objectives:**

This presentation will describe our experience of bringing together clinical and non-clinical staff from two separate organisations to deliver a more appropriate, comprehensive and timely telehealth service to patients. It will explain how strong working relationships have developed, the importance of a clear understanding of different roles within the team and the need for building trust and confidence in colleagues, resulting in the clinical nurse specialists ‘letting go’ and responding to change that supports effective monitoring and still providing quality care. We will report on the lessons learned during the process, from both staff groups’ perspectives and the patient’s perspective, as tasks previously undertaken by clinicians have shifted to non-clinical staff.

**Results:**

Our current approach to telehealth has evolved into a model which ensures that the specialist nursing team are able to focus solely on delivering quality clinical care enabled and supported by telehealth where appropriate. All the non-clinical tasks are now undertaken by the telecare team staff and chronic conditions management administrative support and include:

- Installing devices in patients’ homes and providing education and training
- First-line monitoring/triage of uploaded patient data Escalation of clinical alerts to nursing team by Telephone
- Resolving technical alerts and missing uploads/data
- Provision of refresher training to patients as required (telephone-based or face-to-face)
- Responding to patient or nurse-reported technical problems, including battery/faulty device replacement
- Providing advice on home set-up e.g., recommending changes
- System administrator, patient administration and management function of backend

The results of patient and staff questionnaires seeking feedback on our model will be given together with an economic evaluation comparing the current approach, which utilises telecare and specialist staff to deliver the service compared to the previous delivery model using specialist nursing staff only. We will also show that through embedding telehealth into a well-established community specialist nursing service has the following impact and outcomes:

- Patients to take more responsibility for their day-to-day care
- Nurses to monitor patients remotely and contact those who need support reducing the number if inappropriate home visits
- Reducing travelling for the nursing service
- Improving relationships between patient and nurse
- Supporting carers

**Conclusions:**

We have embedded our telehealth service into existing service models which have now been enhanced utilising a partnership approach and ensuring the best use of the skills and expertise, across the organisations involved. This has been a key factor in developing an efficient, effective and sustainable approach.

